# A Coumarin–Hemicyanine Deep Red Dye with a Large Stokes Shift for the Fluorescence Detection and Naked-Eye Recognition of Cyanide

**DOI:** 10.3390/molecules29030618

**Published:** 2024-01-27

**Authors:** Dongmei Li, Senlin Peng, Xu Zhou, Lingyi Shen, Xianjiong Yang, Hong Xu, Carl Redshaw, Chunlin Zhang, Qilong Zhang

**Affiliations:** 1 School of Basic Medical Science, Guizhou Medical University, Guiyang 550004, China; 15508509190@163.com (D.L.); pengsenlinyuna@outlook.com (S.P.); zhouxu200003@126.com (X.Z.); shenly@stumail.nwu.edu.cn (L.S.); yangxianjiong@126.com (X.Y.); zcl@gmc.edu.cn (C.Z.); 2 Chemistry, School of Natural Sciences, University of Hull, Cottingham Road, Hull HU6 7RX, UK; c.redshaw@hull.ac.uk

**Keywords:** coumarin–hemicyanine, deep red fluorescent dye, large Stokes shift, cyanide

## Abstract

In this study, we synthesized a coumarin–hemicyanine-based deep red fluorescent dye that exhibits an intramolecular charge transfer (ICT). The probe had a large Stokes shift of 287 nm and a large molar absorption coefficient (ε = 7.5 × 10^5^ L·mol^−1^·cm^−1^) and is best described as a deep red luminescent fluorescent probe with *λ*_em_ = 667 nm. The color of probe W changed significantly when it encountered cyanide ions (CN^−^). The absorption peak (585 nm) decreased gradually, and the absorption peak (428 nm) increased gradually, so that cyanide (CN^−^) could be identified by the naked eye. Moreover, an obvious fluorescence change was evident before and after the reaction under irradiation using 365 nm UV light. The maximum emission peak (667 nm) decreased gradually, whilst the emission peak (495 nm) increased gradually, which allowed for the proportional fluorescence detection of cyanide (CN^−^). Using fluorescence spectrometry, the fluorescent probe W could linearly detect CN^−^ over the concentration range of 1–9 μM (*R*^2^ = 9913, RSD = 0.534) with a detection limit of 0.24 μM. Using UV-Vis spectrophotometry, the linear detection range for CN^−^ was found to be 1–27 μM (*R*^2^ = 0.99583, RSD = 0.675) with a detection limit of 0.13 μM. The sensing mechanism was confirmed by ^1^H NMR spectroscopic titrations, ^13^C NMR spectroscopy, X-ray crystallographic analysis and HRMS. The recognition and detection of CN^−^ by probe W was characterized by a rapid response, high selectivity, and high sensitivity. Therefore, this probe provides a convenient, effective and economical method for synthesizing and detecting cyanide efficiently and sensitively.

## 1. Introduction

Anion recognition has become one of the most important parts of supramolecular chemistry due to the vital roles played by anions in the environment, chemistry and biology. Additionally, anions have potential applications in sensors, functional materials and transmembrane transport [1]. Among the various anions, cyanide has received widespread attention, as it is involved in the synthesis of fibers, resins and herbicides, electroplating and gold extraction processes [2,3,4,5].

Cyanide is a highly toxic substance, and trace amounts of cyanide can be absorbed through the gastrointestinal tract, lungs and skin, leading to lesions and issues within the human respiratory system and central nervous system. Moreover, since the brain tissue is extremely sensitive to oxygen, it is the first to be damaged, which ultimately leads to tissue toxicity hypoxia asphyxiation and loss of consciousness and ultimately death [6]. According to the Analytical Methods of Grain Hygiene Standards, the detection limit of cyanide in grain was determined to be 0.015 mg/kg, and the detection limit of cyanide in the Hygiene Standards for Drinking Water for Domestic Purposes (GB5749-2006) [7] was set at 0.07 mg/L. According to the literature, the lethal dose of cyanide for adults is 0.5–3.5 mg. Cyanide has a wide range of sources and can be released from natural substances such as cassava, almonds and some fruit seeds, in which it mainly exists in the form of cyanogenic glycoside ligands, such as tapioca glucoside in cassava and bitter amygdalin in bitter almonds [8,9]. These substances produce hydrocyanic acid under the catalytic action of enzymes in the human body, and the cyanogen ions (CN^−^) produced by them are the root cause of poisoning. The mechanism of action is the inhibition of cytochrome oxidase, so that the cytochrome system is dysfunctional, which leads to asphyxiation of the organism. Therefore, it is essential to monitor and detect the concentration of cyanide in the environment and in humans in real time.

Currently, conventional methods for the detection of cyanide include spectrometry, chromatography and electrochemical methods [10,11,12,13]. However, these methods have the disadvantage of requiring a troublesome and time-consuming pre-treatment of samples, and some of them also rely on expensive instrumentation for detection and have certain limitations in terms of reaction speed and quantification. Fluorescent probes have received widespread attention due to their high sensitivity, speed and environmental compatibility [14,15].

To date, there have been many types of cyanide ion probes reported in the literature, which are mainly classified as bonded fluorescent probes and reactive fluorescent probes, all of which have shortcomings. Although bonded fluorescent probes [16,17] do have the advantages of fast response speeds and good water solubility, they exhibit poor selectivity and are susceptible to the interference of other anions during testing, which is problematic for real-world samples. Reactive fluorescent probes can specifically recognize cyanide without interference from other analytes and congeners, and many fluorescent probes have been developed that can specifically recognize cyanide. However, this type of fluorescent probe is often limited to use in pure organic solvents, and some suffer from slower response speeds [14]. Therefore, the development of new fluorescent probes that can quickly, accurately and efficiently recognize and detect cyanide in real samples is an important research direction.

Fluorescent probes with intramolecular charge transfer (ICT) for the rapid identification and detection of cyanide have been reported in the literature [18,19]. They mainly utilize the nucleophilic addition reaction of the probe with CN^−^, which interrupts the ICT action of the probe, leading to the alteration of the photophysical properties of the probe [20,21,22,23]. Guo et al. [24], obtained a hybrid coumarin–hemicyanine dye with an ICT effect by reacting 1-methyl-2,3,3-trimethyl-3H-indolium and diethylaminocoumarin-aldehyde. The fluorescent probe recognized CN^−^ in MeOH-Tris-HCl buffer (10 mM, pH = 9.3, 1:1, *v*/*v*) with a high selectivity and with proportional fluorescence detection of CN^−^ and a detection limit of 0.6 μM. Considering that most of the anion recognition was carried out in an aqueous solution, the fluorescent probe was clearly water-soluble. Increasing the water solubility of fluorescent probes is necessary, however, and introducing sulfonate groups into the side chain of the molecule is an effective strategy to increase the water solubility of the molecule [25]. In addition, it is also necessary to increase the sensitivity of the probe. Based on this, we reacted diethylaminocoumarin-aldehyde with 4-(2′,3′,3′-trimethylindole-1-quaternary ammonium salt)-butane-1-sulfonate to obtain an ICT-effective coumarin–hemicyanine deep red dye. Due to the introduction of sulfonate into the probe, the water solubility of the probe was increased, and it was able to recognize CN^−^ in dimethyl sulfoxide/PBS (10 mM PBS, pH = 7.40, 1:4, *v*/*v*) with a high selectivity, and the fluorescence detection limit was 0.24 μM, and the UV-Vis detection limit was 0.13 μM.

Furthermore, the designed probe has the following advantages: (1) the probe has a large Stokes shift; (2) the coumarin–semialanthocyanine dye as a chromophore has a large molar absorption coefficient and a large emission wavelength, which can produce obvious signals and color changes after interacting with CN^−^ ions for proportional fluorescence detection and naked-eye recognition; (3) the cationic quaternary ammonium salt as an activating group can accelerate the interaction of cyanide ions with the recognition group (C=N double bond); and (4) the introduction of a sulfonic acid group and a quaternary ammonium salt can increase the solubility of the probe molecule in aqueous media, which is favorable for applications in real samples.

## 2. Results

### 2.1. Structural Characterization

**Figure 1:**
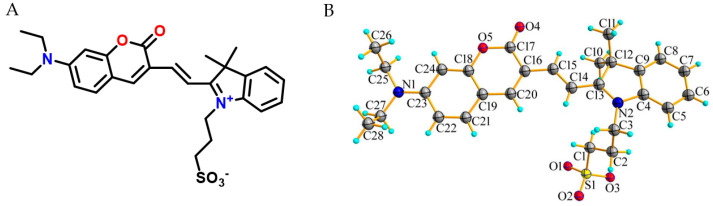
(**A**) Chemical structure of probe W; (**B**) thermal ellipsoid plot at 30% level of the asymmetric units of the complexes W.

The structure of probe W was characterized by ^1^H and ^13^C NMR spectroscopy and HRMS. We successfully obtained crystals of probe W and performed a single-crystal X-ray diffraction experiment. The crystal structure of the probe showed that double bonds (*cis* configuration) connect the coumarin and indole to form an electron donor (D)–π–electron acceptor (A)-type structure. The nitrogen atoms on the ring of the indole already formed a quaternary ammonium salt (coumarin contains a diethylamino group), and this has a strong electron-pushing effect, so the probe has an intramolecular charge transfer (ICT) property and an electron effect. Probe W is dark green in color, with a maximum emission wavelength of 667 nm, i.e., a deep red luminescent probe, with a molar absorption coefficient of ε = 7.5 × 10^5^ L·mol^−1^·cm^−1^ and a Stokes shift of 287 nm. The coumarin and indole rings are not parallel, and they have a dihedral angle of 13.70° between them, whilst the probe molecules interact with each other to form a dimer through typical hydrogen bonding interactions (C25–H25B···O4, C24–H24···O4). Furthermore, the dimer forms typical hydrogen bonding interactions at C14–H14···O2, C3–H3A···O2, C1–H1B···O2 with C14, C3 and C11 of an adjacent dimer, respectively, through the O2 atoms of the sulfonate root (hydrogen bonding parameters are provided in Supplementary Table S2), thus expanding into a one-dimensional structure (Figure 1).

### 2.2. Determination of Optimal Experimental Conditions

If the probe can recognize cyanide in an aqueous solution, this will increase its application value. In addition, the pH of the aqueous solution can be controlled by using a buffer solution to exclude the effect of pH, thus making the identification more accurate and reliable. The optimum water content of the probe used in this experiment was determined experimentally.

**Figure 2:**
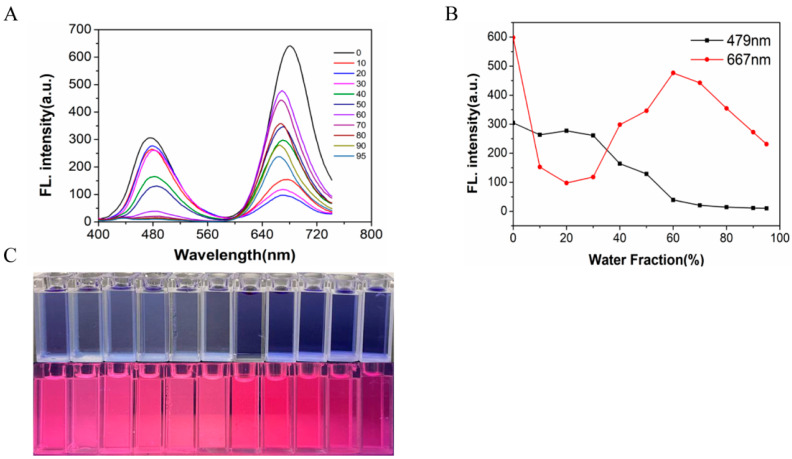
(**A**) Fluorescence spectra of probe W (10 μM) using different water contents; (**B**) effect of water volume fraction (DMSO/PBS system) on fluorescent probe W at maximum emission *λ*_em_ = 667 nm, *λ*_ex_ = 380 nm, slit: 5/5 nm, voltage: 700 V; fluorescence intensity maps of different water volume fractions at 479 nm/667 nm; (**C**) fluorescence intensity maps of different water volume fractions under natural light and changes in fluorescence of different water volume fractions under 365 nm UV irradiation.

The compound showed two emission peaks in dimethyl sulfoxide solution. Starting with blue light emission for *λ*_em_ = 479 nm, the fluorescence intensity did not change significantly as the water content (FW) increased from 0 to 30% and gradually decreased from 30% to 95%. Secondly, the other emission peak was a deep red emission peak of *λ*_max em_ = 667 nm, and the fluorescence intensity decreased significantly when the water content increased from 0 to 20%, and the fluorescence intensity gradually increased to reach the maximum from 20% to 60% and finally decreased again upon increasing the water content to 95% (Figure 2). It emitted red light under irradiation using 365 nm UV light, and a similar phenomenon could be seen in the fluorescence spectrum. Since the fluorescent probe W is insoluble in water and was not sensitive to the recognition and detection of ions or molecules when the water content was high, a mixture of dimethyl sulfoxide/PBS (10 mM PBS, pH = 7.40, 1:4, *v*/*v*) was chosen as the recognition environment.

The pH is also a key parameter that affects the selectivity, sensitivity and detection limit of the probe. Therefore, we experimentally investigated the fluorescence and UV-Vis absorption spectra of probe W over the pH range from 1 to 14.

**Figure 3:**
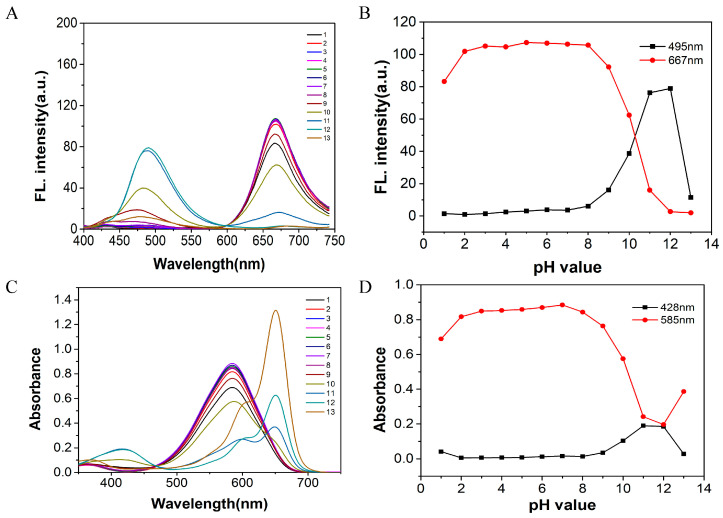
Fluorescence spectra (**A**) of probe W (10 μM) in the assay system of dimethyl sulfoxide/PBS buffer (10 mM PBS, 1:4, *v*/*v*) with different pH values; (**B**) the effect of different pH on the fluorescence of fluorescent probe W at *λ*_em_ = 489 nm/667 nm, *λ*_ex_ = 380 nm, slit: 5/5 nm, voltage: 700 V; (**C**) UV-Vis spectra; (**D**) the effect of different pH on the absorbance of UV-Vis light of fluorescent probe W at *λ* = 428 nm/585 nm.

Firstly, 2.40 mL of PBS buffer of different pH values was added to a 3.00 mL cuvette. Then, 30 μL of the probe stock solution (1 mM) was added to it, and finally the volume of the solution was made up to 3.00 mL using DMSO, shaken well and left to stand until the system was fully reacted. The effects of the different pH values on probe W were detected by use of a fluorescence spectrophotometer and a UV-Vis spectrophotometer. The detection system consisted of dimethyl sulfoxide/PBS (10 mM PBS, 1:4, *v*/*v*), and the fluorescence intensities at *λ*_ex_ = 479 nm and *λ*_em_ = 667 nm tended to stabilize, as did the UV-Vis absorption spectra at 428 nm/585 nm, when in the range from pH = 2 to pH = 8 (Figure 3).

In this experiment, we explored the influence of the water content and pH on the recognition and detection ability of probe W. Dimethyl sulfoxide/PBS buffer (10 mM PBS, pH = 7.40, 1:4, *v*/*v*) was chosen as the detection system.

The optical stability of probe W-CN^−^ (Supplementary Figure S3) was evaluated over time, and the results showed that the fluorescence spectra and UV-Vis absorption spectra were basically unchanged, indicating that probe W was stable. The fluorescence at *λ*_em_ = 495 nm increased rapidly to reach a maximum when probe W-CN^−^ reacted from 0 min to 20 min, and it thereafter remained basically stable upon prolonging the time. The UV-Vis absorption spectra also exhibited the same behavior, so it was concluded that the optimal reaction time for the recognition of CN^−^ by probe W was 20 min.

### 2.3. Anion Sensing Study

A high selectivity and sensitivity of probe W are important parameters for performing water sample detection and in vivo studies. Therefore, in order to test its ability to recognize anions, probe W (10 μM) was combined with a variety of anions (such as Br^−^, C_2_O_4_^2−^, CH_3_COO^−^, Cl^−^, CN^−^, F^−^, H_2_PO_4_^−^, HCO_3_^−^, HPO_4_^2−^, HSO_3_^−^, I^−^, NO_2_^−^, NO_3_^−^, PO_4_^3−^, S^2−^, S_2_O_3_^2−^, SO_3_^2−^ and SO_4_^2−^), cations (such as Al^3+^, Cd^2+^, Fe^3+^, Li^+^, Mg^2+^, Pb^2+^, Zn^2+^, Hg^2+^ and Ag^+^) and amino-containing small molecules (such as GSH and Cys) in dimethyl sulfoxide/PBS buffer (10 mM PBS, pH = 7.40, 1:4, *v*/*v*).

**Figure 4:**
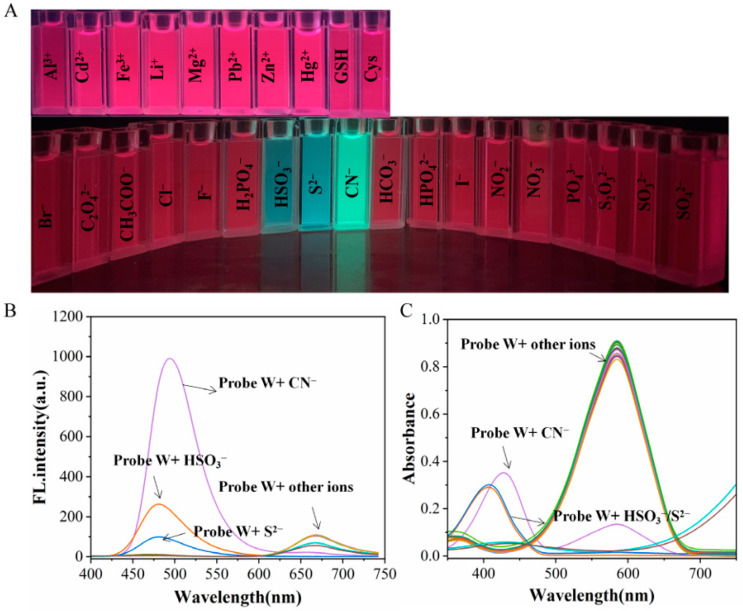
(**A**) Color changes after addition of different anions and cations under 365 nm UV irradiation; (**B**) fluorescence spectra of solutions after addition of different anions and cations to probe W (10 μM) solution in dimethyl sulfoxide/PBS buffer (10 mM PBS, pH = 7.40, 1:4, *v*/*v*) detection system; (**C**) *λ*_ex_/*λ*_em_ = 380 nm/667 nm slit: 5/5 nm, voltage: 670 V and UV-Vis absorption spectrum.

After adding anions to the solution containing probe W, a clear change in the color of the solution was observed under irradiation using 365 nm ultraviolet UV light with CN^−^, HSO_3_^−^ and S^2−^. The fluorescence intensity of CN^−^ was seen to be significantly stronger than that of the other two ions (Figure 4A). The fluorescence spectra also showed that the intensity of the fluorescence emission peak at *λ*_em_ = 495 nm was also significantly stronger when CN^−^ was added versus the addition of either HSO_3_^−^ or S^2−^ (Figure 4B). When fluorescent probe W detected anions, the fluorescence intensity of CN^−^ was stronger than that of HSO_3_^−^ or S^2−^ at *λ*_ex_ = 380 nm. This was because HSO_3_^−^ or S^2−^ are associated with the C=C nucleophilic addition with probe W, whereas CN^−^ is associated with a C=N nucleophilic addition reaction. Moreover, fluorescence probes can usually be visualized and employed for detection in a 365 nm UV dark box. Compared with the existing literature, the different recognition results for the different ions may be due to the different excitation wavelengths employed [26].

In the UV-Vis spectrum, the maximum absorption peak at *λ* = 585 nm decreased significantly after the addition of CN^−^, and a new absorption peak appeared at *λ* = 428 nm. When HSO_3_^−^ and S^2−^ were added to the solution, the maximum absorption peak at *λ* = 585 nm decreased significantly, and a new absorption peak appeared at *λ* = 408 nm (Figure 4C). The above experimental results revealed that probe W can selectively recognize and detect anions such as CN^−^, HSO_3_^−^ and S^2−^ in the UV-Vis spectra. The fluorescence spectra under excitation at *λ*_ex_ = 380 nm and the observation under irradiation using 365 nm UV light revealed that probe W can selectively recognize CN^−^ with a very good selectivity.

**Figure 5:**
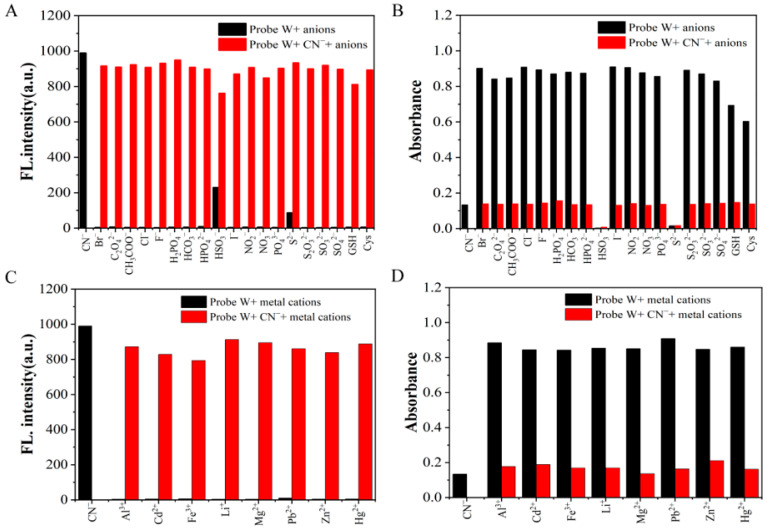
Bar graphs of competition experiments of various anions on (**A**) fluorescence intensity and (**B**) absorbance of probe W-CN^−^ mixtures *λ*_ex_/*λ*_em_ = 380 nm/667 nm, slit: 5/5 nm, voltage: 670 V). Competition experiment bar graphs of (**C**) fluorescence intensity and (**D**) absorbance of various cations against probe W-CN^−^mixture (*λ*_ex_/*λ*_em_ = 380 nm/667 nm, slit: 5/5 nm, voltage: 670 V).

In addition, in order to investigate the competitiveness of probe W in the presence of other competing ions, interference experiments were performed. By observing the change in the fluorescence intensity at *λ*_em_ = 495 nm and absorbance at the absorption peak at *λ*_ex_ = 585 nm and comparing the solution with and without CN^−^, it was found that the fluorescence intensity was higher than that of the other anions when HSO_3_^−^ and S^2−^ were added. Moreover, the absorbance in the UV absorption spectra was lower than that of the other anions, but there was no obvious change in the fluorescence intensity and absorbance upon the addition of other anions and metal cations. The results showed that the coexisting anions and cations had less of an effect on the detection of cyanide. Therefore, the results of the interference experiments showed that probe W has a high specificity, strong selectivity and strong anti-interference ability for the detection of CN^−^ (Figure 5).

### 2.4. Titration and Detection Limits

**Figure 6:**
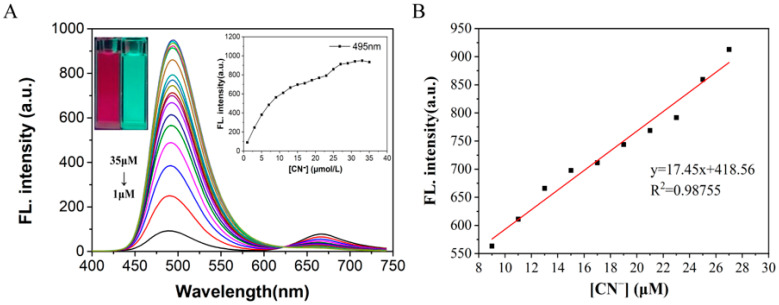
(**A**) Fluorescence spectra of probe W (10 μM) with the addition of CN^−^ and (**B**) linear curves of fluorescence intensity at *λ*_em_ = 495 nm and cyanide concentration (9–27 μM) of the probe solution. Fluorescence intensity curves at *λ*_em_ = 495 nm for different concentrations of cyanide and a photograph of fluorescence color change after irradiation with a 365 nm UV lamp upon completion of the titration (inset in (**A**)).

On the basis of the above experiments, CN^−^ was gradually added to the solution for fluorescence titration experiments. The fluorescence intensity of probe W at *λ*_em_ = 495 nm gradually increased with the increase in the concentration of added CN^−^ ions and finally reached a steady state (Figure 6). In addition, when the concentration of probe W varied between 1–9 μM, a good linear relationship with the concentration of CN^−^ ions (y = 59.188x + 57.327, *R*^2^ = 0.9913, RSD = 0.534) was observed. In this experiment, the experimental data of the fluorescence titration were utilized for the calculation of the detection limit according to the IUPAC method, i.e., 10 sets of blank samples without CN^−^ were tested under the same conditions, and then the standard deviation (SD) was calculated by the fluorescence intensity at *λ*_em_ = 495 nm. Finally, according to the formula detection limit = 3SD/S (S is the linear slope in the fluorescence titration experiments), it was calculated that the detection limit of probe W for CN^−^ is 0.24 μM. Compared with other CN^−^ detection probes, probe W has a lower detection limit (Supplementary Table S3).

**Figure 7:**
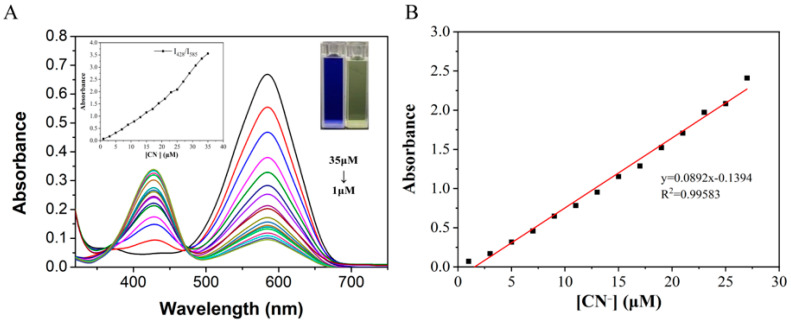
(**A**) UV-Vis absorption spectra of probe W (10 μM) added with CN^−^; (**B**) linear curves of absorbance at 428 nm–585 nm of probe solution and cyanide concentration (1–27 μM). Change curve of absorbance ratio at 428 nm–585 nm for different concentrations of cyanide and a photograph of the solution under ambient light illumination showing the changes in the solution upon completion of the titration (inset in (**A**)).

On the basis of the above experiments, UV titration experiments were carried out by gradually adding CN^−^ to the solution. The absorbance at *λ* = 585 nm in the UV-Vis spectrum decreased, and the absorbance at *λ* = 428 nm increased with the increase in the concentration of added CN^−^ ions (Figure 7). In addition, when the concentration of probe W varied between 1–27 μM, it showed a good linear relationship with the concentration of CN^−^ ions (y = 0.0892x + 0.1394, *R*^2^ = 0.99583, RSD = 0.675). The experimental data from the UV titration were utilized for the calculation of the detection limit by the IUPAC method (as above for the ratio of the absorption peaks at *λ* = 495 nm and *λ* = 585 nm). Finally, according to the formula detection limit = 3SD/S (S is the linear slope in the UV titration experiment), the detection limit of probe W for CN^−^ was calculated as 0.13 μM.

### 2.5. Theoretical Calculations and the Possible Mechanism for Detection of CN^−^

**Figure 8:**
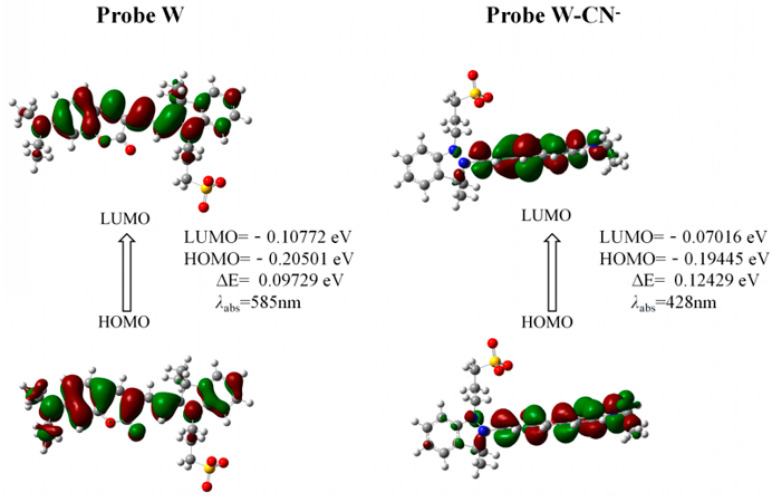
HOMO and LUMO distribution of W and W-CN^−^ calculated by TD-DFT.

**Figure 9:**
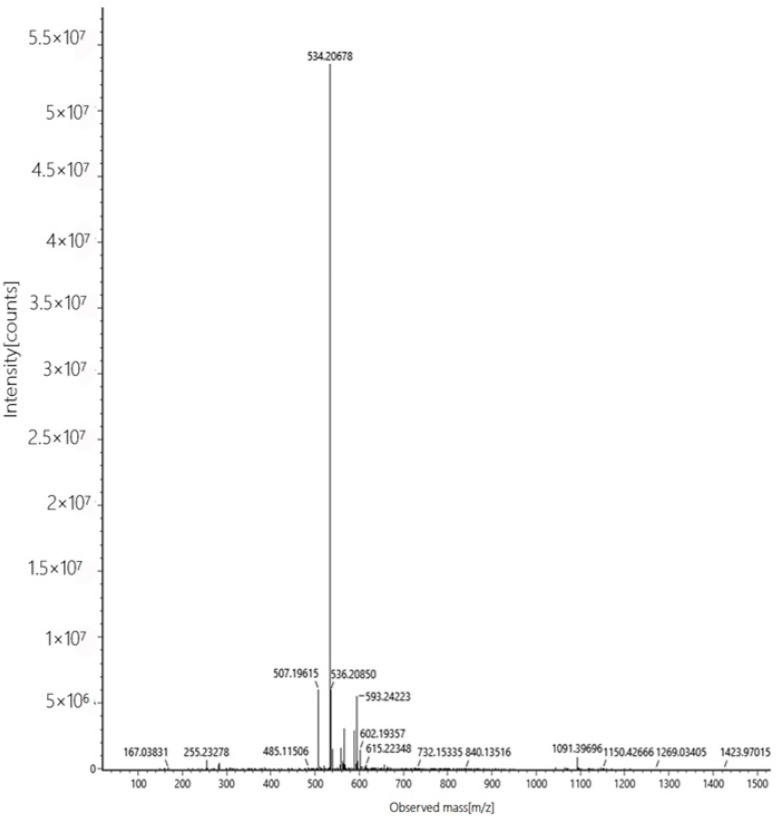
High-resolution mass spectra (HRMS) of the reaction product of probe W upon the addition of CN^−^.

**Figure 10:**
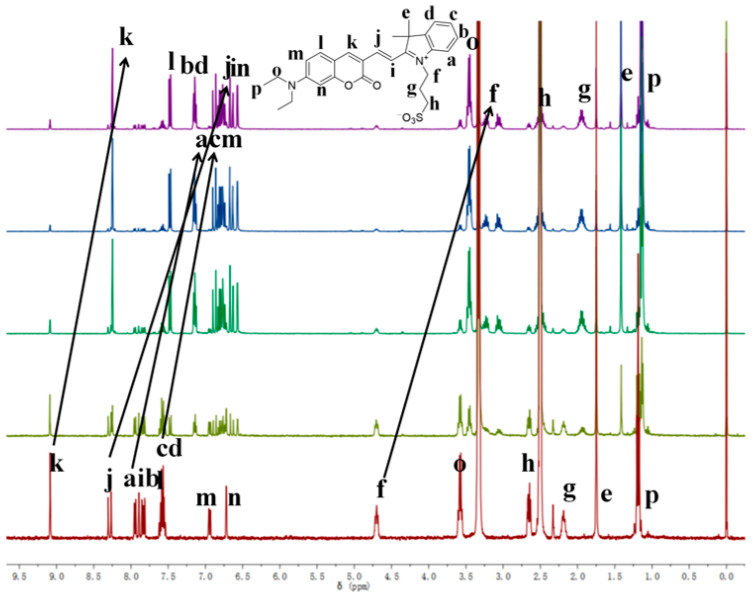
NMR spectral titration of probe W.

**Figure 11:**
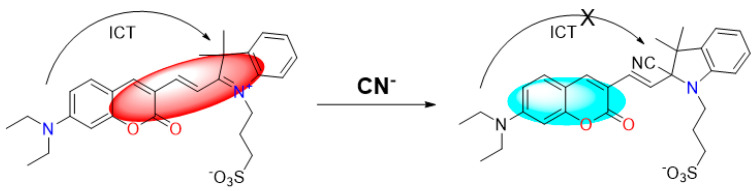
Proposed sensing mechanism of probe W for CN^−^.

Based on the existing literature reports and combining the above experimental results, we speculate here on the reaction mechanism of probe W toward recognizing CN^−^. Probe W contains a C=N double bond, which can easily undergo a nucleophilic addition reaction with CN^−^. The color of the probe changed from blue-violet to colorless due to the addition of CN^−^ to the double bond, destroying the D-π-A structure of the probe and weakening the ICT effect. Quantum chemical calculations of the ground and excited states of probe W and its CN^−^ adducts were carried out using density-functional theory (DFT) and time-varying density-functional theory (TD-DFT), respectively, at the B3LYP/6-311G(d,p) level of theory. The HOMO-LUMO gap of W was 0.09729 eV, whereas the HOMO-LUMO energy level of W-CN^−^ was 0.12429 eV, indicating that the addition of CN^−^ hinders the intramolecular charge transfer. The UV-Vis absorption spectra showed that the absorption peak of W was blue-shifted from 585 nm to 428 nm after the addition of CN^−^, which was consistent with the calculated results (Figure 8). The reaction in the solution between probe W and CN^−^ was verified by high-resolution mass spectrometry (Figure 9). For [C_29_H_32_N_3_O_5_S]^−^: the theoretical value was 534.2068, and the measured value was 534.2067. Subsequently, the CN^−^-sensing mechanism of probe W was investigated by NMR spectroscopic titration experiments (Figure 10). The results of the titration showed that the nucleophilic addition of probe W with CN^−^ generated a neutral compound (Figure 11), which led to a decrease in the electron-absorbing capacity of the indole ring, destroying the conjugated system and inhibiting the ICT process. All the proton signal peaks of probe W were shifted to the high field. In particular, Hk shifted from 9.09 ppm to 8.24 ppm, the proton signal peaks of Hj shifted from 8.29 ppm and 8.27 ppm to 6.90 ppm and 6.85 ppm, the proton signal peaks of Hi shifted from 7.89 ppm and 7.85 ppm to 6.67 ppm and 6.63 ppm and the proton signal peaks of Hf shifted from the triplet peak of 4.68–4.72 ppm to multiplets at 3.02–3.09 ppm. These NMR spectroscopic titration results indicated that a nucleophilic addition reaction between probe W and CN^−^ had occurred.

### 2.6. Applications

One smooth-surfaced and one sprouted green potato were purchased separately and set aside. A total of 0.0800 g of NaOH was added to a beaker containing 20 mL of distilled water and stirred until completely dissolved. A total of 10.00 g of roots and green parts around sprouted potato sprouts were weighed, crushed with a mortar and pestle and transferred to the above NaOH-filled beaker, and distilled water was added to bring the volume up to 30 mL. Then, the system was stirred at 350 rpm for 24 h on a magnetic stirrer protected from light. The stirred solution was removed and centrifuged at 5000 rpm for 30 min, and the supernatant was removed with a 0.22 μm microporous filter to remove large particles that produce fluorescence-dispersed light or absorb light to obtain a cyanide-containing potato solution. A sprouted potato solution containing cyanide was obtained. An unsprouted potato solution was prepared in the same way as above.

The measured fluorescence intensity was applied to the standard curve to obtain the CN^−^ content in the samples and the spiked recoveries. The results in Table 1 show that the spiked recoveries were within the range for both the unsprouted and sprouted potatoes. This indicates that the synthesized fluorescent probe W can be feasibly used to detect CN^−^ in real samples.

## 3. Materials and Methods

### 3.1. Equipment and Reagents

The following equipment items were employed for characterization: Model VGT-2227QTD Ultrasonic Instrument (Shenzhen Guttehongye Machinery Equipment Co., Ltd., Shenzhen, China); Inova-400 MHz NMR Spectrometer (Varian Company, Palo Alto, CA, USA); CP214 electronic balance (Shanghai Aohaus Instrument Co., Ltd., Shanghai, China); Cary Eclipse-type fluorescence spectrophotometer (Varian Company, Palo Alto, CA, USA), UV-Vis spectrophotometer of UV-2600 (Suzhou Dao Jin Instrument Co., Ltd., Suzhou, China) and pHS-25 pH meter (Chengdu Century Ark Technology Co., Ltd., Chengdu, China).

The following chemicals were employed for the synthetic work: Dimethyl sulfoxide (DMSO), anhydrous ethanol (EtOH), cyanide (as KCN), hydrochloric acid (HCl), metal ions, anions and other reagents were purchased from Aladdin Reagent Co., Ltd. (Shanghai, China). All the chemicals were analytically pure, and no further purification was required during use. The water used to configure the solutions during the experiments was ultrapure water (conductivity 18.2 MΩ cm, U-Pure Ultrapure Technology Co., Ltd., Chengdu, China).

### 3.2. Synthesis of Fluorescent Probe W

**Figure 12:**
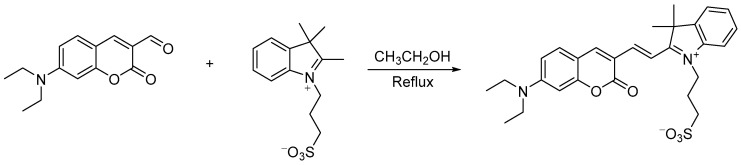
Synthesis route of probe W.

((*E*)-3-(2-(2-(6-(diethylamino)-3-oxo-3,4-dihydronaphthalen-2-yl)vinyl)-3,3-dimethyl-3*H*-indol-1-ium-1-yl)propane-1-sulfonate) was synthesized using a one-step process. The synthesis route is shown in Figure 12:

A total of 100 mg (0.5 mM) of diethylaminocoumarin-aldehyde was dissolved with 232 mg (0.55 mM) of 4-(2′,3′,3′-trimethylindole-1-quaternary ammonium salt)-butane-1-sulfonate in 5 mL of anhydrous ethanol, to which 2 drops of piperidine were added, and the reaction was refluxed for 4 h. The reaction was cooled down to room temperature, and a large amount of dark green solid precipitated, which was pump-filtered, and the solid was washed with 5 mL of anhydrous ethanol 3 times. The obtained solid was recrystallized from 10 mL of methanol, and, finally, the dark green solid probe W was obtained. The molecular formula of fluorescent probe W is C_29_H_32_N_3_O_5_S. ^1^H NMR (400 MHz, DMSO-d_6_) *δ* 9.09 (s, 1H, Pyran-H), 8.29–8.27 (d, *J* = 16.0 Hz, 1H, CH=CH), 7.94 (d, *J* = 7.6 Hz, 1H, Ar-H), 7.89–7.85 (d, *J* = 19.1, 11.6 Hz, 1H, CH=CH), 7.84–7.82 (d, *J* = 16.0 Hz, 1H, Ar-H), 7.64–7.51 (m, 3H, Ar-H), 6.94 (dd, *J* = 9.1, 2.4 Hz, 1H, Ar-H), 6.72 (d, *J* = 2.1 Hz, 1H, Ar-H), 4.72–4.68 (m, 2H, N^+^-CH_2_), 3.57 (q, *J* = 7.1 Hz, 4H, N-CH_2_), 2.64 (s, 2H, -CH_2_SO_3_^−^), 2.19 (s, 2H, -CH_2_), 1.75 (s, 6H, -CH_3_), 1.18 (t, *J* = 7.0 Hz, 6H, -CH_3_) (Supplementary Figure S1). ^13^C NMR (151 MHz, DMSO-*d*_6_) *δ* 179.99, 161.38, 158.90, 154.71, 150.53, 142.71, 140.92, 135.13, 129.54, 128.53, 122.66, 113.32, 113.27, 111.59, 111.21, 108.61, 97.04, 97.02, 51.34, 46.93, 45.75, 45.05, 27.65, 24.73, 12.63 (Supplementary Figure S2). HRMS calculated: 534.2068, found: 534.2067.

### 3.3. X-ray Crystallography

An APEX 2 CCD diffractometer with graphite-monochromated Mo Kα radiation (*λ* = 0.71073 Å) in the ω scan mode was used [27]. The structure was solved by a charge-flipping algorithm and refined by full-matrix least-squares methods on F2 [28]. All esds were estimated using the full covariance matrix. Further details are presented in Supplementary Table S1. CCDC: 2307327.

### 3.4. Preparation of Fluorescent Probe W and Ion Stock Solution

A total of 5.08 mg of the probe was weighed and dissolved in 10 mL of DMSO to prepare a probe stock solution at a concentration of 1 mM. The probe was used as a stock solution for the preparation of the probe. The metal ions of nitrate and the sodium salts of anions (Al^3+^, Cd^2+^, Fe^3+^, Li^+^, Mg^2+^, Pb^2+^, Zn^2+^, Hg^2+^, Ag^+^, Br^−^, C_2_O_4_^2−^, CH_3_COO^−^, Cl^−^, CN^−^, F^−^, H_2_PO_4_^−^, HCO_3_^−^, HPO_4_^2−^, HSO_3_^−^, I^−^, NO_2_^−^, NO_3_^−^, PO_4_^3−^, S^2−^, S_2_O_3_^2−^, SO_3_^2−^ and SO_4_^2−^) were accurately weighed and dissolved in PBS to prepare an ionic reserve solution at a concentration of 10 mM. The PBS solution was prepared by accurately weighing 23.00 g of PBS powder and dissolving it in 2 L of ultrapure water at pH 7.20–7.40.

## 4. Conclusions

In summary, in this study, a one-step method was used to synthesize the target compound probe W using 7-diethylamino-3-formylcoumarin and 4-(2′, 3′, 3′-trimethylindole-1-quaternary ammonium)-butane-1-sulfonate. Probe W has a large Stokes shift, a large molar absorbance coefficient and a large emission wavelength, and it belongs to the family of deep red luminescence luminescent fluorescent probes. Probe W is capable of producing obvious fluorescent signal changes and color changes upon interacting with CN^−^ ions, achieving proportional fluorescence detection and naked-eye recognition. The detection limit using the fluorescence spectrum was 0.81 μM and with a UV-Vis spectra value of 0.13 μM. In addition, probe W was applied to the detection of CN^−^ in samples of germinated potatoes and showed good results. In this experiment, a probe that can detect CN^−^ efficiently and sensitively was synthesized, and an experimental method for the detection of CN^−^ was established.

## Figures and Tables

**Table 1:** Detection of CN^−^ in unsprouted and sprouted portions of potatoes by probe W.

Sample	Measured Concentration after Spiking (μM)	Sample Concentration (μM)	Spiked Concentration (μM)	Spiked Recovery Rate (%)
Unsprouted potato	2.64	0.65	2.00	99.62
	4.77		4.00	102.58
	6.39		6.00	96.09
Sprouted potato	2.61	0.68	2.00	97.39
	4.92		4.00	105.13
	6.88		6.00	102.99

## Data Availability

The datasets presented in this study can be found in online repositories. The names of the repository/repositories and accession number(s) can be found below: https://www.ccdc.cam.ac.uk/ accessed on 24 January 2024, CCDC: 2307327.

## Supplementary Materials

The following supporting information can be downloaded at: https://www.mdpi.com/article/10.3390/molecules29030618/s1, Figure S1: ^1^H NMR spectrum of probe W; Figure S2: ^13^C NMR spectrum of probe W; Figure S3: Time-varying fluorescence spectra (A) of probe W-CN^−^ solution in dimethyl sulfoxide/water (V_DMSO_:V_H2O_ = 1:4, 10 mM PBS buffer) detection system; changes in fluorescence intensities of probe W-CN^−^ solution over time at the maximum emission at *λ*_em_ = 495 nm, slit. 5/5 nm, voltage: 700 V (B); UV-Vis absorption spectra (C) and the effect of time-dependent UV-Vis absorbance of probe W-CN^−^ solution at *λ* = 428/585 nm (D); Table S1: Summary of crystal data for W; Table S2: Hydrogen-bonding geometries of compound W; Table S3: Comparison data with reported CN^−^ sensors. References [23,29,30,31,32,33,34,35] are cited in the supplementary materials.
