# Supplementary material for: A Coumarin–Hemicyanine Deep Red Dye with a Large Stokes Shift for the Fluorescence Detection and Naked-Eye Recognition of Cyanide
Source: Molecules. 2024 Jan 27;29(3):618. doi: 10.3390/molecules29030618 (PMC10856579; doi:10.3390/molecules29030618)
Supplement: Supplementary file 1 [file molecules-29-00618-s001.zip › molecules-2834347-supplementary.pdf]

A Coumarin-Hemicyanine Near-Infrared Dye with a Large Stokes Shift for the Fluorescence Detection of Cyanide and Naked-Eye Recognition

Dongmei Li[1], Senlin Peng[1], Xu Zhou[1], Lingyi Shen[1], Xianjiong Yang[1], Hong Xu[1,*], Carl Redshaw[2], Qilong Zhang[1,*]

1 School of Basic Medical Science, Guizhou Medical University, Guiyang 550004, China. Email: shenly@stumail.nwu.edu.cn (L.Y.S); sciqlzhang@gmc.edu.cn (Q.L.Z) ; yangxianjiong@126.com (X.J.Y); ylh6401@gmc.edu.cn (Y.L.H); xuhong@gmc.edu.cn (H.X.).

2 Chemistry, School of Natural Sciences, University of Hull, Cottingham Road, Hull, Yorkshire HU6 7RX, UK. mail: c.redshaw@hull.ac.uk (C.R.).

* Correspondence: xuhong@gmc.edu.cn (H.X.); Fax: +86-851-8817-4017 (H.X.); gzuqlzhang@126.com (Q.L.Z.); Fax: +86-851-8817-4017 (H.X.)

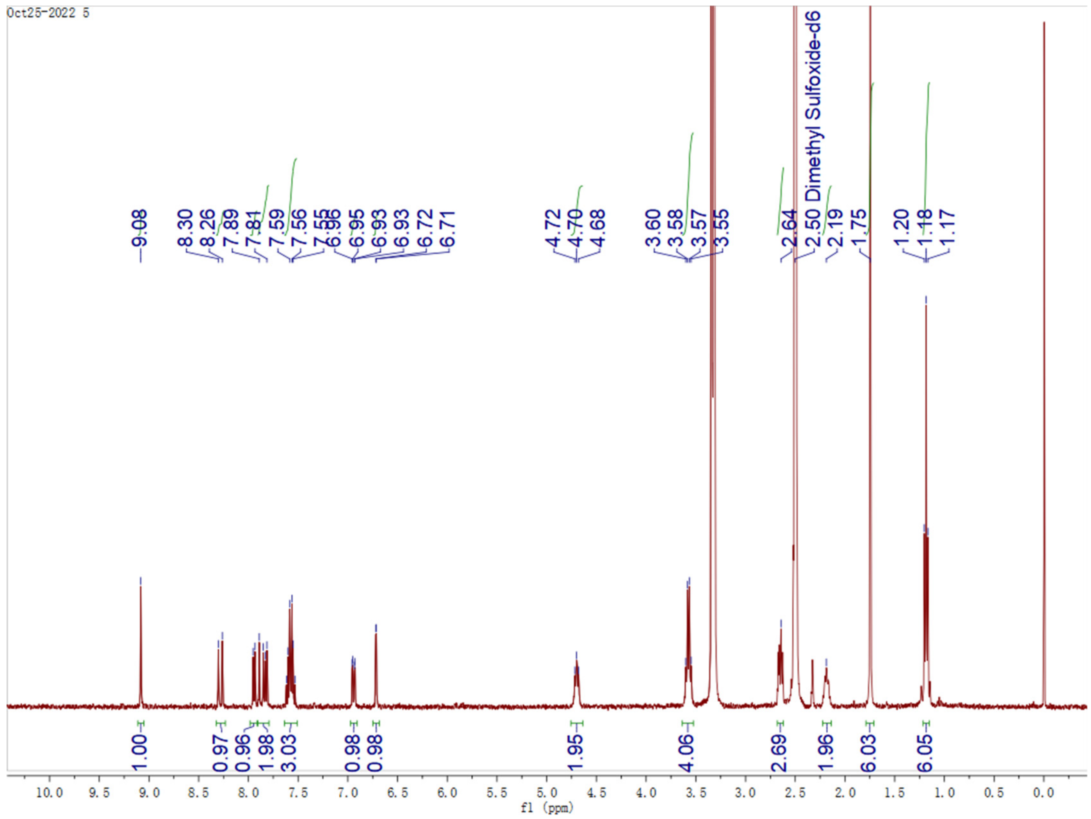

Figure S1. ^{1}H NMR spectrum of probe W

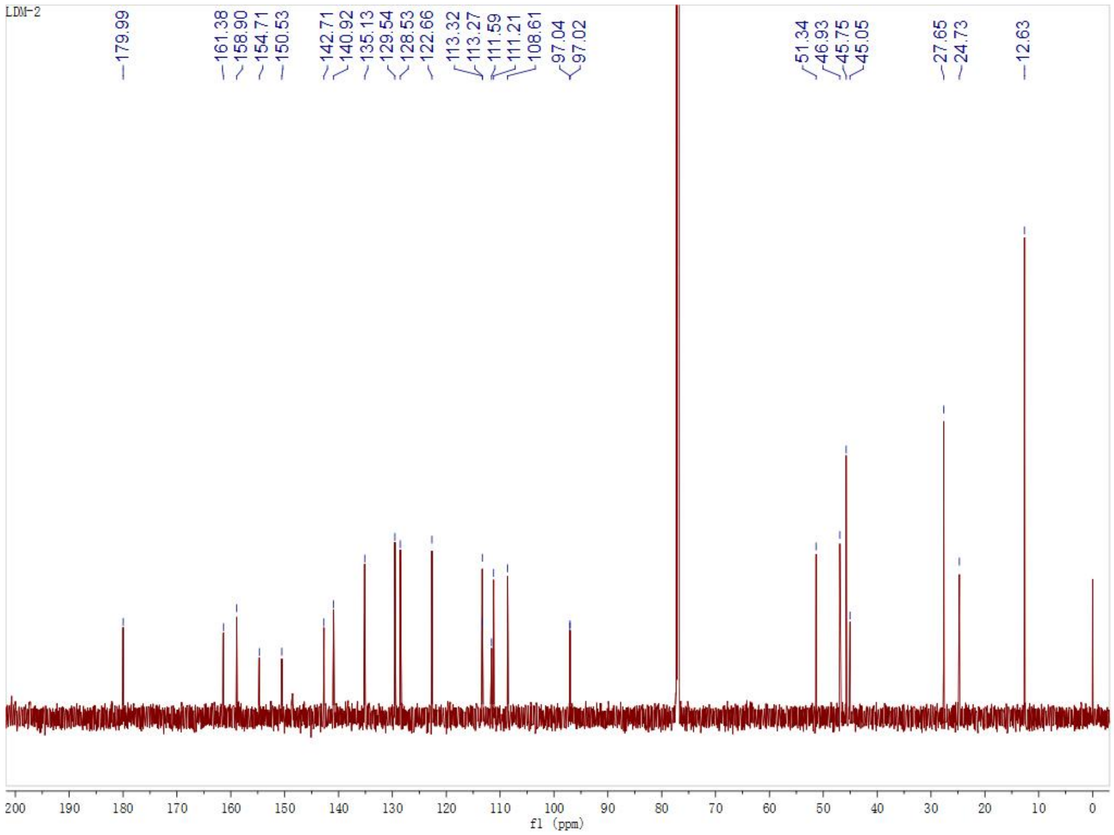

Figure S2. ^{13}C NMR spectrum of probe W

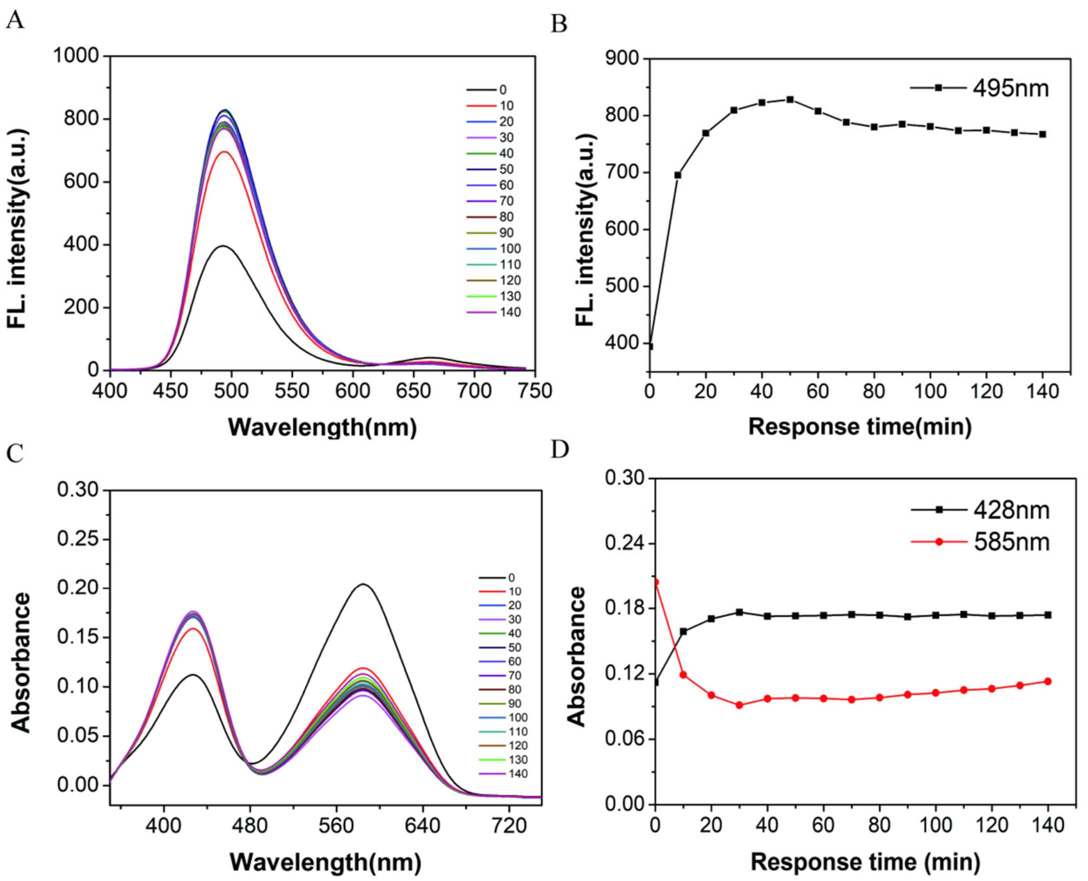

Figure S3. Time-varying fluorescence spectra (A) of probe W-CN^-solution in dimethyl sulfoxide/water (V_{DMSO}:V_{H2O}=1:4, 1.00×10^{-2}mol/L PBS buffer) detection system; changes in fluorescence intensities of probe W-CN^-solution over time at the maximum emission at λ_{em}=495nm, slit. 5/5nm, voltage: 700V (B); UV-Vis absorption spectra (C) and the effect of time-dependent UV-visible absorbance of probe W-CN^-solution at λ=428/585nm (D).

Table S1. Summary of crystal data for W

Parameter	W
Empirical formula	$C_{28}H_{32}N_2O_5S$
Formula weight [g mol^{-1}]	508.61
Crystal system	monoclinic
Space group	$P2_1/n$
a [Å]	16.317(3)
b [Å]	7.8841(17)
c [Å]	20.827(4)
α	90.00°
β	104.707(9)
γ	90.00°
Volume [Å^3]	2591.6(9)
Z	4
Density, calcd [gm^{-3}]	1.304
Temperature [K]	300(2)
Unique reflns	2463
Obsd reflns	4564
Parameters	330
R_{int}	0.0774
$R[I>2\sigma(I)]^a$	0.0745
W[all data]R^b	0.1679
GOF on F^2	1.060

Table S2. Hydrogen-bonding geometries for compound W

D–H···A	d(D···H)/nm	d(H···A)/nm	d(D···A)/nm	∠D–H···A/ (°)
C(10)-H(10B)-F(3) ^{i}	0.096	0.254	0.329 9(4)	137
C(10)-H(10B)-F(4) ^{i}	0.096	0.243	0.337 8(4)	170
C(3)-H(3)-F(1) ^{ii}	0.093	0.254	0.343 0(4)	161
C(10)-H(10C)-O(2) ^{iii}	0.096	0.257	0.337 9(4)	141
C(18)-H(18A)-F(4) ^{iii}	0.097	0.251	0.331 3(4)	140
C(19)-H(19B)-F(2)	0.096	0.250	0.338 6(4)	153
C25—H25B···O4 ^{i}	0.97	2.40	3.242 (6)	144.6
C24—H24···O4 ^{i}	0.93	2.40	3.306 (5)	164.7
C14—H14···O2 ^{ii}	0.93	2.43	3.348 (6)	171.0
C3—H3A···O2 ^{ii}	0.97	2.62	3.458 (6)	145.0
C1—H1B···O2 ^{ii}	0.97	2.48	3.347 (6)	149.4

Symmetry codes: i −x+1, −y, −z+1; ii x−1, y, z; iii x, −y+1/2, z+1

Table S3. Comparison data with reported CN^- sensors

Structure	Fluorescence	solvent	Detection limit
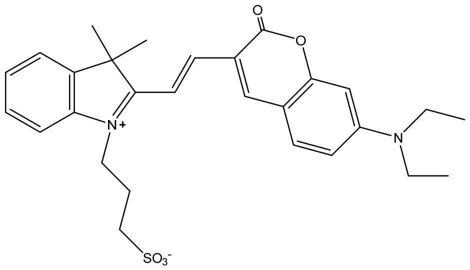 **Our work**	Turn-on	DMSO/ H_2O (1:4)	0.24 μM/ 0.13 μM
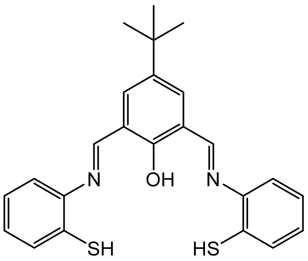 **Probe R [2]**	Turn-on	DMSO/ H_2O (2:8)	0.96 μM
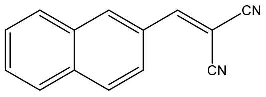 **Probe 1 [3]**	Turn-on	DMF/H_2O (6:4)	0.23 μM
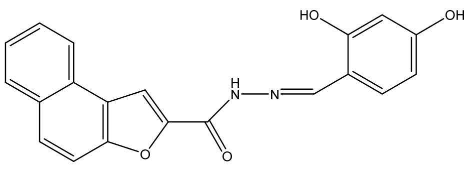 **Q1-3 [4]**	Turn-on	DMSO/ H_2O (1:9)	0.81 μM

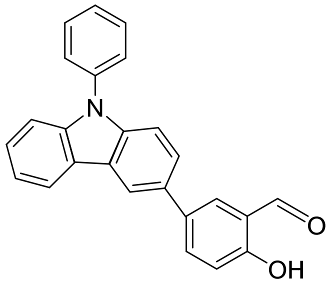 **CPHB [5]**	Turn-on	THF/ H_2O (1:19)	0.249 μM
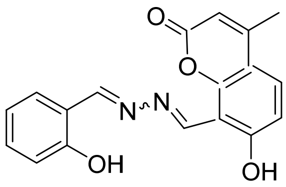 **YS [6]**	Turn-on	DMSO/ H_2O (3:7)	0.17 μM
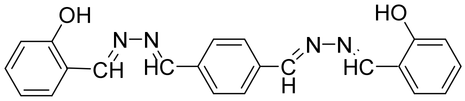 **L1 [7]**	Turn-on	DMSO/ H_2O (4:1)	0.947 μM
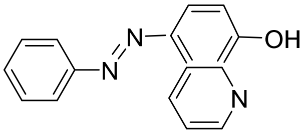 **AZQ [8]**	Turn-on	EtOH/ H_2O (1:1)	2.6μM

References

1. Shi, Q; Wu, S.T; Shen, L.Y; Zhou, T; Xu, H; Wang, Z.Y; Yang, X.J; Huang, Y.L; Zhang, Q.L. A Turn-On Fluorescent Chemosensor for Cyanide Ion Detection in Real Water Samples. *Frontiers in chemistry*. 2022, 10:923149. doi:10.3389/fchem.2022.923149.
2. Kumar, P.S; Lakshmi, P.R; Elango, K.P. An easy to make chemoreceptor for the selective ratiometric fluorescent detection of cyanide in aqueous solution and in food materials. *New Journal of Chemistry*. 2019, 43(2), 675-680. doi:10.1039/C8NJ05587D.
3. Long, L.L; Yuan, X.Q; Cao, S.Y; Han, Y.Y; Liu, W.G; Chen, Q; Han, Z.X; Wang, K. Determination of Cyanide in Water and Food Samples Using an Efficient Naphthalene-Based Ratiometric Fluorescent Probe. *ACS Omega*. 2019, 4(6), 10784–10790. doi:10.1021/acsomega.9b01308.
4. Qu, W.J; Li, W.T; Zhang, H.L; Wei, T.B; Lin, Q; Yao, H; Zhang, Y.M. Rapid and Selective Detection of Cyanide Anion by Enhanced Fluorescent Emission and Colorimetric Color Changes at Micromole Levels in Aqueous Medium. *Journal of Heterocyclic Chemistry*. 2018, 155-124. doi:10.1002/jhet.3113.
5. Zhang, Y.X; Lu, L.Y; Pan, J.M; Liu, H; Ma, J. Preparation a novel phenylcarbazole-based fluorescent sensor for the sensitive and selective detection of CN− in water. *Materials Letters*. 2022, 313. DOI:10.1016/j.matlet.2022.131745.
6. Hu, J.H; Sun, Y; Qi, J; Li, Q; Wei, T.B. A new unsymmetrical azine derivative based on coumarin group as dual-modal sensor for CN^- and fluorescent "OFF-ON" for Zn^{2+}. *Spectrochimica acta. Part A, Molecular and biomolecular spectroscopy*. 2017, 175:125-133. doi:10.1016/j.saa.2016.12.009.
7. Pei, P.X; Hu, J.H; Chen, Y; Sun, Y; Qi. J. A novel dual-channel chemosensor for CN^- using asymmetric double-azine derivatives in aqueous media and its application in bitter almond. *Spectrochimica Acta Part A: Molecular and Biomolecular Spectroscopy*. 2017, 181:131-136. doi: 10.1016/j.saa.2017.03.052.
8. Koc, O.K; Ozay, H. A simple azoquinoline based highly selective colorimetric sensor for CN^- anion in aqueous media. *Canadian Journal of Chemistry*. 2017, 0163–. doi:10.1139/cjc-2017-0163.
